# *Drosophila melanogaster White* Mutant *w*^*1118*^ Undergo Retinal Degeneration

**DOI:** 10.3389/fnins.2017.00732

**Published:** 2018-01-04

**Authors:** María José Ferreiro, Coralia Pérez, Mariana Marchesano, Santiago Ruiz, Angel Caputi, Pedro Aguilera, Rosa Barrio, Rafael Cantera

**Affiliations:** ^1^Departamento de Biología del Neurodesarrollo, Instituto de Investigaciones Biológicas Clemente Estable, Montevideo, Uruguay; ^2^Center of Cooperative Research in Biosciences CIC bioGUNE, Bizkaia Technology Park, Derio, Spain; ^3^Departamento de Neurociencias Integrativas y Computacionales, Instituto de Investigaciones Biológicas Clemente Estable, Montevideo, Uruguay; ^4^Zoology Department, Stockholm University, Stockholm, Sweden

**Keywords:** *Drosophila*, transgenic lines construction, reporter gene, *white* mutation, neurodegeneration

## Abstract

Key scientific discoveries have resulted from genetic studies of *Drosophila melanogaster*, using a multitude of transgenic fly strains, the majority of which are constructed in a genetic background containing mutations in the *white* gene. Here we report that *white* mutant flies from *w*^*1118*^ strain undergo retinal degeneration. We observed also that *w*^*1118*^ mutants have progressive loss of climbing ability, shortened life span, as well as impaired resistance to various forms of stress. Retinal degeneration was abolished by transgenic expression of *mini-white*^+^ in the *white* null background *w*^*1118*^. We conclude that beyond the classical eye-color phenotype, mutations in *Drosophila white* gene could impair several biological functions affecting parameters like mobility, life span and stress tolerance. Consequently, we suggest caution and attentiveness during the interpretation of old experiments employing *white* mutant flies and when planning new ones, especially within the research field of neurodegeneration and neuroprotection. We also encourage that the use of *w*^*1118*^ strain as a wild-type control should be avoided.

## Introduction

One landmark of modern genetics can be dated to January 1910, when Thomas Hunt Morgan discovered a male of *Drosophila melanogaster* with white eyes (Morgan, 1910; Green, 1996). In the following 100 years, *white* (*w*^−^) mutant fruitflies became one of the most useful tools for the advancement of genetics and played a fundamental role in modern biology. The invention of stable germline transformation (Rubin and Spradling, 1982) led to the generation of thousands of *Drosophila* transgenic fly lines used to investigate a wide array of biological questions. This technology relies mainly on the use of *w*^−^ mutant embryos for the construction and selection of efficient transformants during the generation of transgenic fly strains (St. Johnston, 2013).

The *Drosophila* gene *w* (CG2759) is a central part of the eye-pigmentation pathway. It encodes an ATP binding cassette transporter, White (O'Hare et al., 1984; Pepling and Mount, 1990), that forms dimers with either Brown or Scarlet proteins, encoded by *brown* and *scarlet* genes respectively. The White-Brown dimer transports guanine (Sullivan et al., 1979) and the White-Scarlet dimer transports tryptophan and kynurenine (Sullivan and Sullivan, 1975), all of which are precursors used for the synthesis of the two eye pigments, drosopterin, and ommochrome (Nolte, 1952). In neurons, these transporters contribute to the synthesis of biogenic amines. Tryptophan is used to synthesize serotonin and guanine is used for the synthesis of biopterin, a co-factor for the synthesis of serotonin and dopamine (Goodwill et al., 1998). Hence, *w*^−^ mutant flies have abnormally low levels of the biogenic amines serotonin, dopamine, and histamine (Borycz et al., 2008; Sitaraman et al., 2008). The *w* gene is expressed principally in eyes, where it accumulates in the membrane of pigment granules (Mackenzie et al., 2000), as well as in excretory organs and testes (Fjose et al., 1984; Pirrotta et al., 1985; Mackenzie et al., 2000; Evans et al., 2008). Very low levels are observed in the glia and neurons of the brain (Borycz et al., 2008) and in various other tissues (Chintapalli et al., 2007).

One of the functions of the fly eye pigment granules is to improve visual acuity through optic isolation of the photosensitive units (rhabdomeres) within each optical unit (ommatidium). Accordingly, *w*^−^ mutant fruitflies kept in standard laboratory conditions have enhanced light sensitivity (Wu and Wong, 1977) but deficient visual acuity (Kalmus, 1943), contrast and brightness (Wehner et al., 1969), as well as other problems (see Belušič, 2011 for review). Another function of the White protein is to protect retinal photoreceptors from excessive exposure to light (Shoup, 1966; Schraermeyer and Dohms, 1993; Lee and Montell, 2004; Bulgakova et al., 2010). More recently, it was discovered that mutations in *w* gene exacerbate the retinal degeneration observed in flies with transgenic expression of human Tau (Ambegaokar and Jackson, 2010). Additional eye-related abnormalities of *w*^−^ mutant flies include abnormal phototaxis and electroretinogram (ERG) (Stark and Wasserman, 1972; Markow and Scavarda, 1977; Wu and Wong, 1977; Kain et al., 2012), and a substantial decrease in the number of synaptic vesicles of photoreceptor terminals (Borycz et al., 2008).

Several studies have shown that mutations in *Drosophila w* gene have also consequences beyond the eye, comprising a variety of neurological phenotypes: changes in male sexual behavior (Zhang and Odenwald, 1995; Anaka et al., 2008; Lee et al., 2008), anesthesia resistance (Campbell and Nash, 2001), variations in the period of locomotion recovery following anoxia (Xiao and Robertson, 2016), strongly reduced aggressive behavior (Hoyer et al., 2008), impaired olfactory and spatial learning (Diegelmann et al., 2006; Anaka et al., 2008; Sitaraman et al., 2008), hypersensitivity to ethanol (Chan et al., 2014) and to certain tactile stimuli (Titlow et al., 2014), among others. In spite of our vast knowledge regarding these neurological phenotypes, *Drosophila w*^−^ mutants are frequently used as “wild-type controls” relative to other mutants or transgenic flies (e.g., Chinchore et al., 2012; Manzanillo et al., 2013; Bulat et al., 2014; Lincoln et al., 2015; Snijder et al., 2015; West et al., 2015; Gupta et al., 2016; Haddadi et al., 2016).

Here we asked whether *w*^−^ mutations cause neurodegeneration. This question arose from several observations. Abnormal levels of *w* transcripts were reported in three genomic studies of neurodegeneration (Scherzer et al., 2003; Shieh and Bonini, 2011; Ferreiro et al., 2012), and mutated *w* was found to enhance *tau*-induced retinal degeneration (Ambegaokar and Jackson, 2010). We applied several assays currently used in *Drosophila* to define neurodegenerative pathologies and found that *w*^−^ mutant flies suffer from an age-dependent, progressive neurodegenerative retinal phenotype.

## Materials and methods

### Stocks and laboratory conditions

Experiments were conducted using *D. melanogaster* males and/or virgin females from *w*^*1118*^*, w*^*1*^ or *mini-w*^+^ on a *w*^*1118*^ background (*w*^*1118*^*;{P[w[*+*mc]* = *UAS-GFP. S65T]}*^*II*^*T*_*10*_) stocks, and two wild-type stocks commonly used by the scientific community, i.e., *Oregon R* (http://flybase.org/reports/FBsn0000276.html) and *Vallecas* (Morata and Garcia-Bellido, 1973), named hereafter as *w*^+^. Flies were raised in standard conditions (25°C, 12:12 h light:dark cycle, standard food). Flies were anesthetized either with nitric oxide (Inject+Matic Sleeper) or CO_2_ for sex identification under a stereoscopic microscope.

### Retinal histology

Histological sections of the retina were prepared from virgin female flies of *w*^*1118*^, *w*^*1*^, *mini-w*^+^ in *w*^*1118*^ background, or *w*^+^ stocks, aged 5, 15, or 30 days. Five flies of each genotype and age were anesthetized and decapitated with a sharp needle. Heads were placed on a microscope slide within a droplet of physiological saline solution. The proboscis was cut off and the occipital cuticle was removed, using fine forceps and a sharp needle, to improve fixative penetration. Heads were fixed overnight in an ice-cold solution of 2.5% glutaraldehyde and 4% paraformaldehyde prepared in 0.1 M phosphate buffered saline pH 7.3. After rinsing in saline solution heads were post-fixed for 1 h in 0.5% osmium tetroxide, rinsed in water, dehydrated in 10 min steps (50, 70, 80, 90, and 100% ethanol and twice in acetone for 20 min), embedded in resin (AGAR 100, AGAR Scientific), and polymerized at 60°C for 48 h. Histological sections of 1 μm thickness were cut with a glass knife on a RMX MT-X ultramicrotome, stained with 0.1% boracic toluidine blue and mounted on microscope slides with DPX (AGAR Scientific) for observation with an Olympus IX81 microscope. Sections were carefully taken at about the same depth/region of the eye to allow proper comparison. Images were acquired with a digital microscope camera Olympus DP71 and processed with Adobe Photoshop.

### Lacunae measurements

Lacunae were quantified in three virgin female flies from *w*^*1118*^ and *w*^+^ stocks, aged 5, 15 and 30 days and in three virgin female flies from *w*^*1*^ stock aged 30 days. We registered lacunae number per genotype and age, measured lacunae area and calculated average lacunae area (μm^2^) for each genotype and age. *w*^+^ flies never showed lacunae. Statistical analyses were conducted using STATISTICA (7.0 Version, StatSoft, Inc.). The Shapiro–Wilk test (Shapiro et al., 1968) was used to check for normal distribution and Levene test (Brown and Forsythe, 1974) was used to check for homogeneity of variances. When both conditions were confirmed, One-Way ANOVA parametric test was used (lacunae number, *w*^*1*^ 30 d vs. *w*^*1118*^ 30 d). If not, non-parametric Kruskal–Wallis (Kruskal and Wallis, 1952) was used instead (lacunae number and area, *w*^*1118*^ 5 d vs. *w*^*1118*^ 15 d vs. *w*^*1118*^ 30 d) and Mann–Whitney *U*-test (Mann and Whitney, 1947) was used for *post-hoc* analysis, or directly Mann–Whitney *U*-test (lacunae area, *w*^*1118*^ 30 d vs. *w*^*1*^ 30 d).

### Rhabdomere measurements

The size (diameter in cross section) of each rhabdomere in photoreceptors R1 to R7 was measured in three virgin female flies from *w*^*1118*^ and *w*^+^ stocks, aged 5 and 30 days. Rhabdomeres were measured in retinal histological sections (one eye per fly, six equatorial located ommatidia per eye). Statistical analyses were conducted using STATISTICA (7.0 Version, StatSoft, Inc.). The Shapiro–Wilk test (Shapiro et al., 1968) was used to check for normal distribution and Levene test (Brown and Forsythe, 1974) to check for homogeneity of variances. After both conditions were confirmed, Two-Way ANOVA parametric test was used to check for significant differences in rhabdomeres R1-R7 diameter between different genotypes of the same age (*w*^+^ 5 d vs. *w*^*1118*^ 5 d and *w*^+^ 30 d vs. *w*^*1118*^ 30 d) and between different ages of the same genotype (*w*^+^ 5 d vs. *w*^+^ 30 d and *w*^*1118*^ 5 d vs. *w*^*1118*^ 30 d). The Fisher exact test or Bonferroni test were used for *post-hoc* analysis. The percentage of ommatidia with seven rhabdomeres (i.e., the total number that can be observed at this level of the retina in normal flies) was calculated from histological sections of three virgin female flies from *w*^*1118*^ and *w*^+^ stocks, aged 5 and 30 days, from three virgin female flies from *w*^*1*^ stock aged 30 days and from three virgin female flies from *mini-w*^+^ stock aged 30 days (about 200 ommatidia per genotype and age). The Shapiro–Wilk test (Shapiro et al., 1968) was used to check for normal distribution and Levene test (Brown and Forsythe, 1974) to check for homogeneity of variances. When both conditions were confirmed, Two-Way ANOVA parametric test was used to check for significant differences in the percentage of ommatidia with seven rhabdomeres between different genotypes of the same age (*w*^+^ 5 d vs. *w*^*1118*^ 5 d and *w*^+^ 30 d vs. *w*^*1118*^ 30 d) and between different ages of the same genotype (*w*^+^ 5 d vs. *w*^+^ 30 d and *w*^*1118*^ 5 d vs. *w*^*1118*^ 30 d). The Fisher exact test or Bonferroni test were used for *post-hoc* analysis. If not, non-parametric Kruskal–Wallis (Kruskal and Wallis, 1952) was used instead (*w*^+^ 30 d vs. *w*^*1118*^ 30 d vs. *w*^*1*^ 30 d). Mann–Whitney *U*-test (Mann and Whitney, 1947) was used for *post-hoc* analysis.

### Electroretinogram

ERG assays were conducted in retinas of live virgin female flies from *w*^*1118*^ and *w*^+^ stocks, aged 5 and 30 days (*n* = 4 per genotype and age). Flies were first immobilized by ice cooling and placed with their heads emerging from the tip of a disposable plastic micropipette, in order to manipulate their orientation under a microscope (Axioscope, Zeiss). After an adaptation period of at least 10 min, ERG recordings were obtained with glass electrodes filled with saline solution. The active electrode was placed on the cornea of the right eye. The reference electrode was a wire inserted in saline-soaked cotton and touching the fly body. The stimulus was a light pulse emitted by a 15 mA white LED placed at 5 cm from the cornea. A dim light background, generated by a computer monitor placed about 1.5 m apart was present during the experiments. The stimulus regime consisted of a train of 50 rectangular pulses of 130 ms each, separated by 5 s. Electrode voltage was amplified using an Axoclamp 2B (Axon Instruments) and continuously sampled at 20 kHz using Pclamp software (Axon Instruments). Post-stimulus recordings from each fly were averaged off-line per genotype and age, and their traces were overlapped for visual comparison. Statistical analyses were done using Willcoxon rank-sum test (Wilcoxon, 1945).

### Climbing assays

For climbing assays, 30–50 virgin male and female flies from *w*^*1118*^ and *w*^+^ stocks (separated per genotype and sex in tubes of 10 flies each), were selected within 1 day after hatching. Flies were transferred every 3–5 days to tubes containing fresh food. Climbing ability was tested in these flies at four times along their life (5, 15, 25, and 30 days of age). Each tube was quickly tapped 8 consecutive times to make the flies fall to the bottom: this forces all flies to start climbing (negative geotaxis reflex, flies move opposite the Earth's gravitational vector when disturbed). Ten seconds later, we recorded the number of flies that have crossed a line drawn at 8 cm from the bottom of the tube. This procedure was repeated 10 times for each tube, leaving a 1 min interval between each measurement. The 10 measurements per tube were averaged for graphical representation and statistic comparisons. All assays were made under red light to avoid phototaxis effects. Data obtained were compiled into Excel tables and plotted per genotype, sex and age. Statistical analyses were conducted using STATISTICA (7.0 Version, StatSoft, Inc.). The Shapiro–Wilk test (Shapiro et al., 1968) was used to check for normal distribution and Levene test (Brown and Forsythe, 1974) to check for homogeneity of variances. When both conditions were confirmed, Two-Way ANOVA parametric test was used to check for significant differences in climbing ability between different genotypes of the same age (females analysis: *w*^+^ 5 d vs. *w*^*1118*^ 5 d, *w*^+^ 15 d vs. *w*^*1118*^ 15 d, *w*^+^ 25 d vs. *w*^*1118*^ 25 d and *w*^+^ 30 d vs. *w*^*1118*^ 30 d) and between different ages of the same genotype (females analysis: *w*^+^ 5 d vs. *w*^+^ 15 d vs. *w*^+^ 25 d vs. *w*^+^ 30 d and *w*^*1118*^ 5 d vs. *w*^*1118*^ 15 d vs. *w*^*1118*^ 25 d vs. *w*^*1118*^ 30 d). The Fisher exact test or Bonferroni test were used for *post-hoc* analysis. If not, non-parametric Kruskal–Wallis (Kruskal and Wallis, 1952) was used instead (males analysis: *w*^+^ 5 d vs. *w*^*1118*^ 5 d, *w*^+^ 15 d vs. *w*^*1118*^ 15 d, *w*^+^ 25 d vs. *w*^*1118*^ 25 d, *w*^+^ 30 d vs. *w*^*1118*^ 30 d, *w*^+^ 5 d vs. *w*^+^ 15 d vs. *w*^+^ 25 d vs. *w*^+^ 30 d, *w*^*1118*^ 5 d vs. *w*^*1118*^ 15 d vs. *w*^*1118*^ 25 d vs. *w*^*1118*^ 30 d). Mann–Whitney *U*-test (Mann and Whitney, 1947) was used for *post-hoc* analysis.

### Stress assays: starvation, sugar-enriched diet, paraquat, and hydrogen peroxide treatments

For stress assays we collected males and virgin female flies from *w*^*1118*^ and *w*^+^ stocks during their first day of life (dextrose assays: *n* = 60 flies per genotype and sex; starvation assay: *n* = 60 flies per genotype and sex; paraquat assays: *n* = 50 flies per genotype, sex and paraquat concentration; hydrogen peroxide assays: *n* = 20 flies per genotype, sex and hydrogen peroxide concentration). Flies of each genotype and sex were kept in tubes containing ~10 flies each. For dextrose treatment, animals were placed into tubes having a filter paper in the bottom soaked in a 5% dextrose-water solution. For starvation experiments, adults were placed into tubes having a piece of paper soaked in water (to avoid thirst and desiccation) but without food. For paraquat and hydrogen peroxide treatments, animals were first starved overnight (Wang et al., 2008). Next morning, animals were placed into tubes having a filter paper soaked in a 5% sucrose-water solution containing either paraquat (2, 10, or 20 mM) or hydrogen peroxide (0.5 or 5%). Half-life was measured for each tube (each corresponding to 10 flies), and used for statistically comparing genotypes of the same sex. Two-Way ANOVA parametric test was used to check for significant differences in half-life between genotypes and treatment conditions. The Fisher exact test or Bonferroni test were used for *post-hoc* analysis.

### Life span measurement

For life span experiments we used males and virgin female flies from *w*^*1118*^ and *w*^+^ stocks selected immediately after hatching. Twenty to hundred flies per genotype and sex were kept in groups of ~10 flies per tube. Flies were transferred every 3–5 days to tubes containing fresh food. For total life span measurement (25°C assays: *n* = 100 flies per genotype and sex; dextrose assays: *n* = 60 flies per genotype and sex; starvation assays: *n* = 60 flies per genotype and sex; paraquat assays: *n* = 50 flies per genotype, sex and paraquat concentration; hydrogen peroxide assays: *n* = 20 flies per genotype, sex and hydrogen peroxide concentration), the number of dead flies per tube was counted every day from day 1 until the last fly died. Half-life was calculated as the age where 50% of the flies of each genotype, sex and experimental condition died. Data obtained were compiled into Excel tables, plotted per genotype and sex, and separated by stress treatment. Statistical analyses were conducted using STATISTICA (7.0 Version, StatSoft, Inc.). The Shapiro–Wilk test (Shapiro et al., 1968) was used to check for normal distribution and Levene test (Brown and Forsythe, 1974) to check for homogeneity of variances. When both conditions were confirmed, Two-Way ANOVA parametric test was used to check for significant differences in half-life between genotypes and treatment conditions. The Fisher exact test or Bonferroni test were used for *post-hoc* analysis.

### Optical neutralization of the cornea

For the analysis of retinal organization in *mini-w*^+^ flies, in addition to histological sections, we used the method of optical neutralization of the cornea as previously described (Franceschini and Kirschfeld, 1971; Franceschini et al., 1981). After nitric oxide anesthesia and decapitation, the heads of 30 days-old *mini-w*^+^ and *w*^+^ flies were mounted on a microscope slide with a droplet of transparent nail polish. Illumination through the eye using an Olympus IX81 inverted microscope allowed to visualize the tips of the rhabdomeres with a 40 x objective. Images were acquired with a digital microscope camera (Olympus DP71) and processed with Adobe Photoshop.

## Results

### Mutations in *Drosophila white* gene cause progressive retinal degeneration

**Figure 1:**
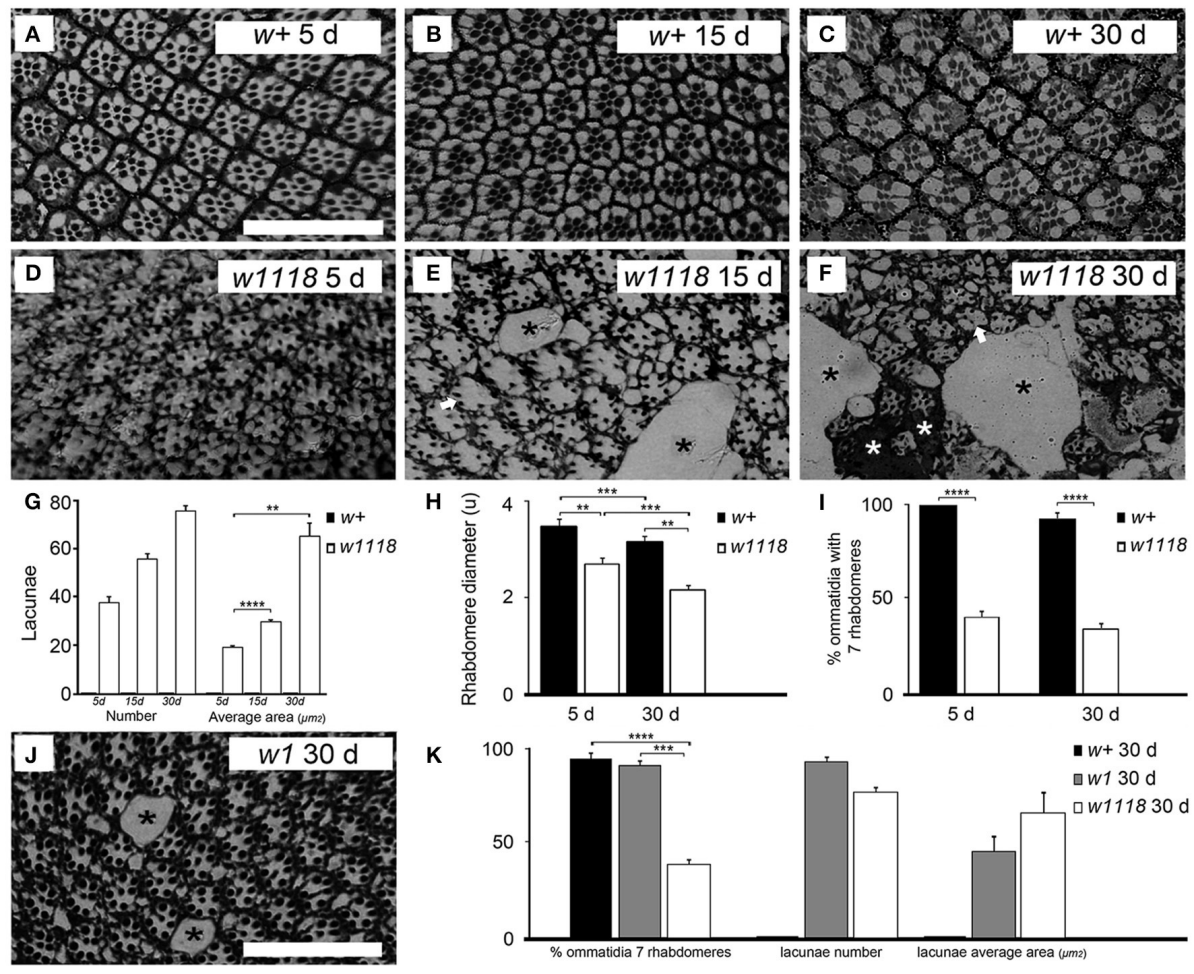
*D. melanogaster w*^*1118*^ mutants show progressive retinal degeneration. Histological microscopy sections of the retina from *w*^+^ flies of the Vallecas strain **(A–C)** and from *w*^*1118*^ mutant flies **(D–F)** showed that *w*^*1118*^ mutants suffer from progressive retinal degeneration. Mild disorganization of the geometrical pattern of ommatidia was detected at 5 days **(D)** and increased at 15 **(E)** and 30 **(F)** days. In these later stages some ommatidia lacked one or more rhabdomeres (white arrows in **E,F**) and others were even entirely missing, leaving empty spaces or lacunae (black stars in **E,F**). In some cases, these empty spaces appeared to be filed by glial cells (white stars in **F**). The scale bar shown in **(A)** represents 40 μm. The same magnification was used in all the panels **(A–F)**. **(G)** Graphical representation of the number and size of lacunae per age and genotype. Lacunae were never present in *w*^+^ flies but were present in *w*^*1118*^ mutants, showing a tendency to increase in number with age (*w*^*1118*^ 5 d vs. 15 d vs. 30 d, Kruskal–Wallis test, *p* > 0.05). The size of lacunae increased with age (Kruskal–Wallis test, Mann–Whitney *U*-test as *post-hoc, w*^*1118*^ 5 vs. 15 d ^****^p < 0.0001, *w*^*1118*^ 5 vs. 30 d ^**^p < 0.01). The bars indicate standard error of the mean (s.e.m.). **(H)** Rhabdomere diameter is expressed as the mean diameter (in μm ± s.e.m.) of rhabdomeres R1 to R7, for 5 and 30 d *w*^+^ (Vallecas) and *w*^*1118*^ females. The size of rhabdomeres R1-R7 was reduced with age in both genotypes (*w*^+^ 5 vs. 30 d and *w*^*1118*^ 5 vs. 30 d, Two-Way ANOVA, Bonferroni *post-hoc*, ^***^p < 0.001 and ^**^p < 0.01). In both ages rhabdomeres were smaller in *w*^*1118*^ compared to *w*^+^ (*w*^*1118*^ vs. *w*^+^ at 5 and 30 d, Two-Way ANOVA; Bonferroni *post-hoc*, ^***^p < 0.001 and ^**^p < 0.01). The bars indicate s.e.m. **(I)** Graphical representation of the percentage of ommatidia that shows seven rhabdomeres in *w*^+^or *w*^*1118*^ females 5 or 30 days-old. *w*^*1118*^ 5 and 30 d mutants had significantly less ommatidia with seven rhabdomeres than *w*^+^ flies of the same ages (*w*^*1118*^ vs. *w*^+^ at 5 and 30 d, *w*^*1118*^ 5 vs. 30 d and *w*^+^ 5 vs. 30 d, Two-Way ANOVA *p* < 0.0001; Fisher test *post-hoc*, ^****^p < 0.0001). There were no significant differences in the number of ommatidia with seven rhabdomeres between different ages within each genotype. The bars indicate s.e.m. **(J)** Histological microscopy sections of the retina from 30 days-old *w*^*1*^ mutants showing a degenerative phenotype of the retina similar to that of *w*^*1118*^ mutants, although with a milder disorganization of the geometrical pattern of ommatidia. Some ommatidia were entirely missing, leaving empty spaces or lacunae (black stars). The scale bar represents 40 μm. **(K)** Contrary to what was observed in *w*^*1118*^ 30 d mutants, *w*^*1*^ 30 d mutants had similar number of ommatidia with seven rhabdomeres than *w*^+^ 30 d flies (*w*^*1118*^ vs. *w*^+^ vs. *w*^*1*^ at 30 d, One-Way ANOVA *p* < 0.0001; Bonferroni *post-hoc*, ^***^p < 0.001, ^****^p < 0.0001). Lacunae were present in both *w*^−^ mutants but never in *w*^+^ flies. *w*^*1*^ 30 d mutants showed similar number and area of lacunae than *w*^*1118*^ 30 d mutants (lacunae number and average area comparison, *w*^*1118*^ 30 d vs. *w*^*1*^ 30 d, One-way ANOVA and Mann–Whitney *U*-test respectively, *p* >0.05). The bars indicate s.e.m.

Retinal degeneration can be precisely monitored in *Drosophila* by examination of histology sections. We prepared sections of the retina from *w*^*1118*^ mutants and *w*^+^ flies aged 5, 15, and 30 days and from *w*^1^ mutants aged 30 days. The extraordinarily regular array of ommatidia in the *w*^+^ fly eye (Figures 1A–C) allows detection of even small deviations from the normal pattern in the *w*^*1118*^ fly eye (Figures 1D–F). A modest but clear phenotype was observed already in the eye of *w*^*1118*^ mutants of the youngest age (5 d), comprising mild disorganization of the characteristic pattern of ommatidia (Figure 1D) and occasional lacunae probably representing missing ommatidia (Figure 1G). This *w*^*1118*^ phenotype, never observed in the retina of *w*^+^ flies, became aggravated with age (15 and 30 d) and comprised greater disorganization and progressively larger lacunae (see black stars in Figures 1E,F and average lacunae area in Figure 1G). In the oldest *w*^*1118*^ flies (30 d), part of the spaces devoid of ommatidia were filled with osmophilic material resembling a glial scar (see white stars in Figure 1F). Lacunae were also observed in 30 d flies from a second mutant *w*^−^ allele (*w*^*1*^) (see black stars in Figures 1J,K).

Retinal degeneration in *Drosophila* is almost always associated with degeneration of the rhabdomeres, i.e., the microvilli-packed apical portion of the photoreceptor enriched in light-sensing proteins (Shieh, 2011). We found that the size of rhabdomeres decreased with age in *w*^+^ and *w*^*1118*^ mutant retinas (5 vs. 30 d). Most importantly, we also found that rhabdomeres from *w*^*1118*^mutants were smaller than those from age-matched *w*^+^ control flies at both ages (Figure 1H). We also quantified the number of ommatidium with seven rhabdomeres in *w*^*1118*^ and *w*^+^ retinas (5 and 30 d), and in 30 d *w*^*1*^ retinas, as seven is the number of rhabdomeres per ommatidium expected to be observed in histological sections taken at this level of the retina (Cagan, 2009). We found significant differences in the percentage of ommatidia with seven rhabdomeres between *w*^*1118*^ and *w*^+^ flies at both ages analyzed. There were no significant age-dependent differences in the percentage of ommatidia with seven rhabdomeres within each genotype. At 5 days of age, all ommatidia contained the complete set of rhabdomeres in *w*^+^ flies but only 46% of ommatidia in *w*^*1118*^ flies. In older flies (30 d) the percentage of ommatidia with seven rhabdomeres was 97% in *w*^+^ flies and 40% in *w*^*1118*^ flies (Figure 1I). *w*^*1*^ 30 d mutants showed a percentage of ommatidia with seven rhabdomeres similar to that of *w*^+^ flies of the same age and differed from that of *w*^*1118*^ 30 d flies (Figure 1K).

Disorganization of the regular array of ommatidia, atrophied rhabdomeres, and progressive loss of photosensitive units will most probably have functional consequences for the retina of *w*^−^ mutants. This can be monitored by ERG, a robust assay applied to a variety of experimental conditions (Belušič, 2011). Previous studies showed that mutations in *Drosophila w* gene affect the ERG in several ways (Stark and Wasserman, 1972; Pak and Lidington, 1974; Wu and Wong, 1977; Belušič, 2011). However, those assays were done with flies of unreported age and/or sex, or carrying additional mutations, making difficult to discern the contribution of sex, age, and genotype to the reported ERG abnormalities.

**Figure 2:**
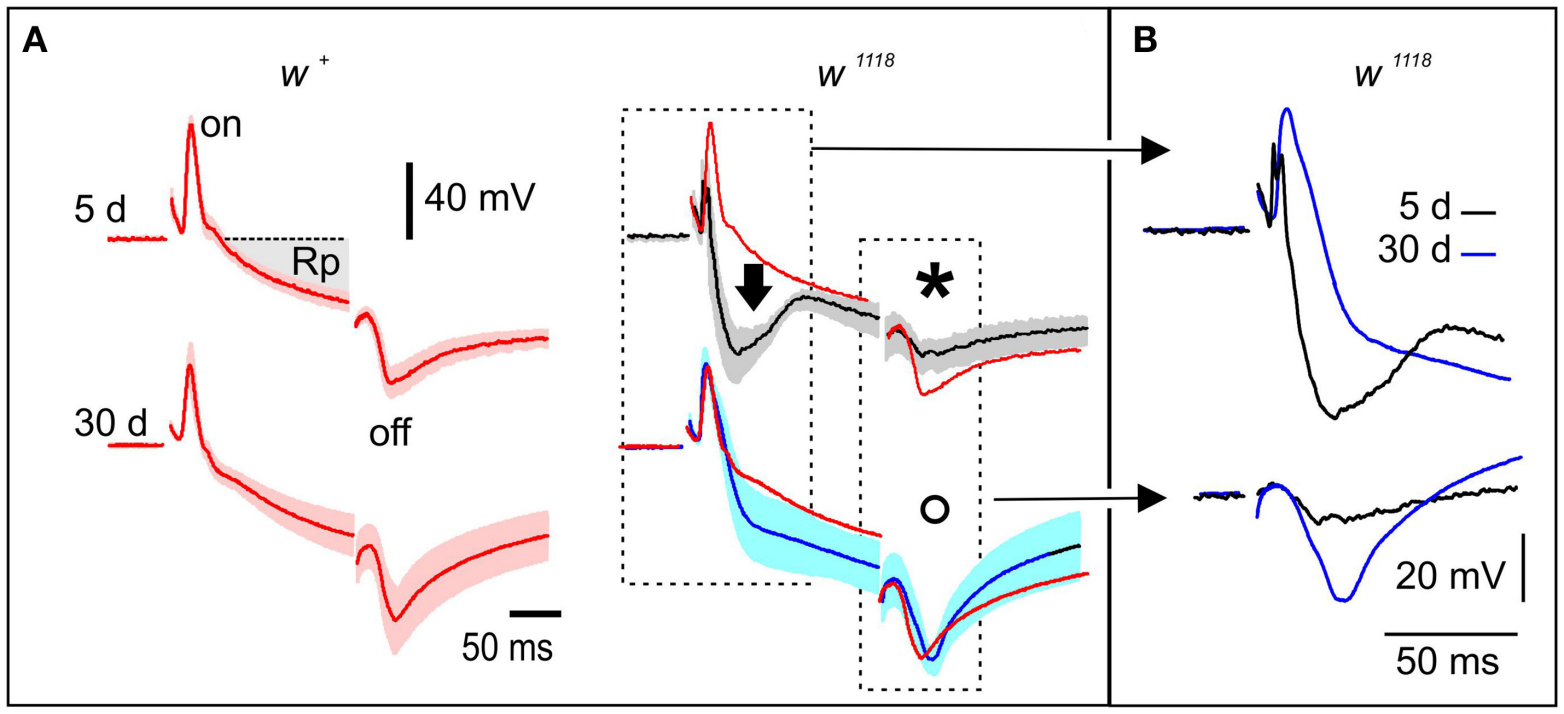
*w*^*1118*^ mutants show abnormal electroretinogram (ERG). **(A)** Each panel shows the mean ERG from four flies. The shadowed area indicates the s.e.m. Two genotypes (*w*^*1118*^ or *w*^+^ flies of the Vallecas strain) and two ages (5 or 30 days) are compared. Different phases of the ERG measured on the surface of the retina are associated with the activation of different structures, the receptor potential (Rp) is generated by the activation of photoreceptors whereas the “on” and “off” potentials are due to the activation of the lamina (Heisenberg, 1971). The black arrow in the right panel (black trace) indicates a massive photoreceptor response characteristic of young *w*^*1118*^ mutants. This response is diminished in 30 days old mutants (right bottom panel, blue trace). The red traces are from young *w*^+^ flies and were included as reference. The amplitude of the “off” potential in 5 days-old mutants is significantly different than in *w*^+^ of the same age (black asterisk, compare top panels) but it is not significantly different at 30 days (open circle, compare bottom panels). **(B)** This panel shows the insets from **(A)** comparing the overall averaged responses in young (5 d) and old (30 d) *w*^*1118*^ mutants showing the main electrophysiological differences due to aging and degeneration on these mutants: the decrease in amplitude of the early photoreceptor response (compare blue with black traces in upper panel) and the recovery of the “off” response (compare blue with black traces in lower panel).

Here we compared the ERG of *w*^*1118*^ and *w*^+^ female flies of 5 and 30 days of age kept in standard laboratory conditions (Figure 2). The ERG of *w*^+^ *Drosophila* flies classically contains three components, the ON potential (on), the receptor potential (Rp), and the OFF potential (off). Rp (shaded gray area Figure 2A top-left) is produced by the activation of photoreceptors while the “on” and the “off” potentials are generated by the synaptic activation of structures present in the lamina (Trujillo-Cenóz, 1965; Heisenberg, 1971). The ERG of *w*^*1118*^ and *w*^+^ flies differed in the shape and time course of the Rp [compare red (*w*^+^) and black (*w*^*1118*^) traces in Figure 2A top right]. This difference is expressed as an initial corneal negativity that opposes (and consequently reduces) the “on” potential and increases the initial stages of Rp in *w*^*1118*^ flies (Figure 2A right, black arrow). This phenomenon is associated with the lack of eye pigments in these mutants as compared to *w*^+^ (reviewed in Belušič, 2011) and is probably due to the massive recruitment of the photoreceptor population in *w*^*1118*^ mutants. We confirmed that these differences between *w*^*1118*^ and *w*^+^ in young (5 d) flies were significant (*p* < 0.05). The comparison of both genotypes at young age showed also that the “off” potential is significantly smaller and delayed in the mutant (Figure 2A, asterisk, compare red and black traces in right top panel; *p* < 0.05). There was no difference in Rp responses between young and old flies of *w*^+^ genotype. In *w*^*1118*^ 30d flies, the Rp difference with *w*^+^ was reduced becoming not statistically significant [Figure 2A, compare red and blue (*w*^*1118*^) traces, *p* = 0.1]. Similarly, the differences in “on” and “off” potentials between *w*^*1118*^ and *w*^+^ were not observed in the 30 d flies (Figure 2A, open circle, compare red and blue traces in right bottom panel). In *w*^+^ flies, the age progression produced a decrease in the early deflection characteristic of the young mutants with a recovery of the “on” and “off” potentials (Figure 2B, compare black and blue traces, *p* < 0.05).

### Mutations in *white* cause deficiencies in locomotion

The majority of neurodegenerative conditions studied in fruitflies and other animals have been associated with deficiencies in locomotor ability (Lessing and Bonini, 2009; Hirth, 2010; Jaiswal et al., 2012). The most frequently used locomotion assay in *Drosophila* is the “climbing assay,” which measures a motor activity that requires a brain circuit including identified neurons expressing the biogenic amine dopamine (Riemensperger et al., 2013).

**Figure 3:**
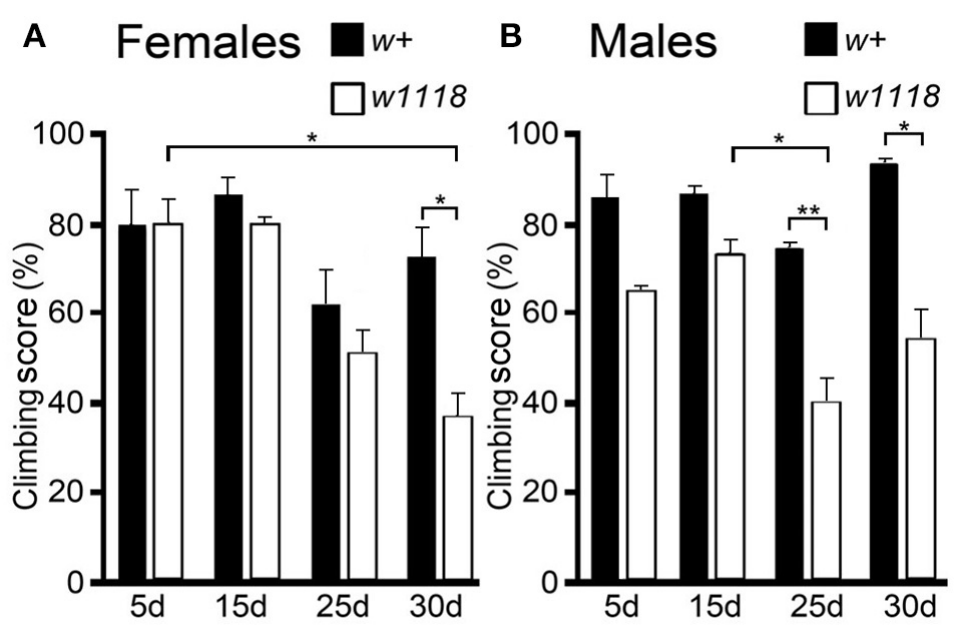
*w*^*1118*^ mutants show progressive deterioration of climbing ability. Scores of climbing assays are expressed as the percentage of flies (**A**, females; **B**, males) from each genotype (*w*^+^ of the Vallecas strain or *w*^*1118*^) that climbed up passed the 8 cm, tested at four different ages (5, 15, 25, and 30 days). The aged mutants had lower climbing scores relative to *w*^+^ irrespective of the sex (*w*^*1118*^ vs. *w*^+^ at 5, 15, 25, and 30 d; *w*^*1118*^ 5 d vs. 15 d vs. 25 d vs. 30 d, and *w*^+^ 5 d vs. 15 d vs. 25 d vs. 30 d; Two-Way ANOVA was used to check for significant differences in female climbing ability between different genotypes of the same age (*w*^+^ 5 d vs. *w*^*1118*^ 5 d, *w*^+^ 15 d vs. *w*^*1118*^ 15 d, *w*^+^ 25 d vs. *w*^*1118*^ 25 d, and *w*^+^ 30 d vs. *w*^*1118*^ 30 d) and between different ages of the same genotype (*w*^+^ 5 d vs. *w*^+^ 15 d vs. *w*^+^ 25 d vs. *w*^+^ 30 d, and *w*^*1118*^ 5 d vs. *w*^*1118*^ 15 d vs. *w*^*1118*^ 25 d vs. *w*^*1118*^ 30 d). The Fisher exact test or Bonferroni test was used for *post-hoc* analysis. Kruskal–Wallis was used for males analysis to compare different genotypes of the same age (*w*^+^ 5 d vs. *w*^*1118*^ 5 d, *w*^+^ 15 d vs. *w*^*1118*^ 15 d, *w*^+^ 25 d vs. *w*^*1118*^ 25 d, *w*^+^ 30 d vs. *w*^*1118*^ 30 d) and to compare different ages of the same genotype (*w*^+^ 5 d vs. *w*^+^ 15 d vs. *w*^+^ 25 d vs. *w*^+^ 30 d, *w*^*1118*^ 5 d vs. *w*^*1118*^ 15 d vs. *w*^*1118*^ 25 d vs. *w*^*1118*^ 30 d). Mann–Whitney *U*-test (Mann and Whitney, 1947) was used for *post-hoc* analysis. ^*^p < 0.05; ^**^p < 0.01. In all cases, the bars indicate s.e.m.

We tested the locomotion ability of *w*^*1118*^ mutant flies using the climbing assay. Flies of either genotype (*w*^*1118*^ or *w*^+^) of both sexes were tested at four different ages from 5 days post-hatching. Thirty days *w*^*1118*^ females had a significant deterioration of their climbing ability relative to *w*^+^ females of the same age and relative to the 5 d *w*^*1118*^ females (Figure 3A). Older *w*^*1118*^ males (25 and 30 d) have a significant deterioration of their climbing ability relative to *w*^+^ males of the same age (Figure 3B). This tendency for lower climbing scores in *w*^*1118*^ flies of both sexes was detected at younger ages, albeit differences with respect to *w*^+^ were not statistically significant (Figures 3A,B).

### Mutations in *white* reduce life span and impair stress resistance

**Figure 4:**
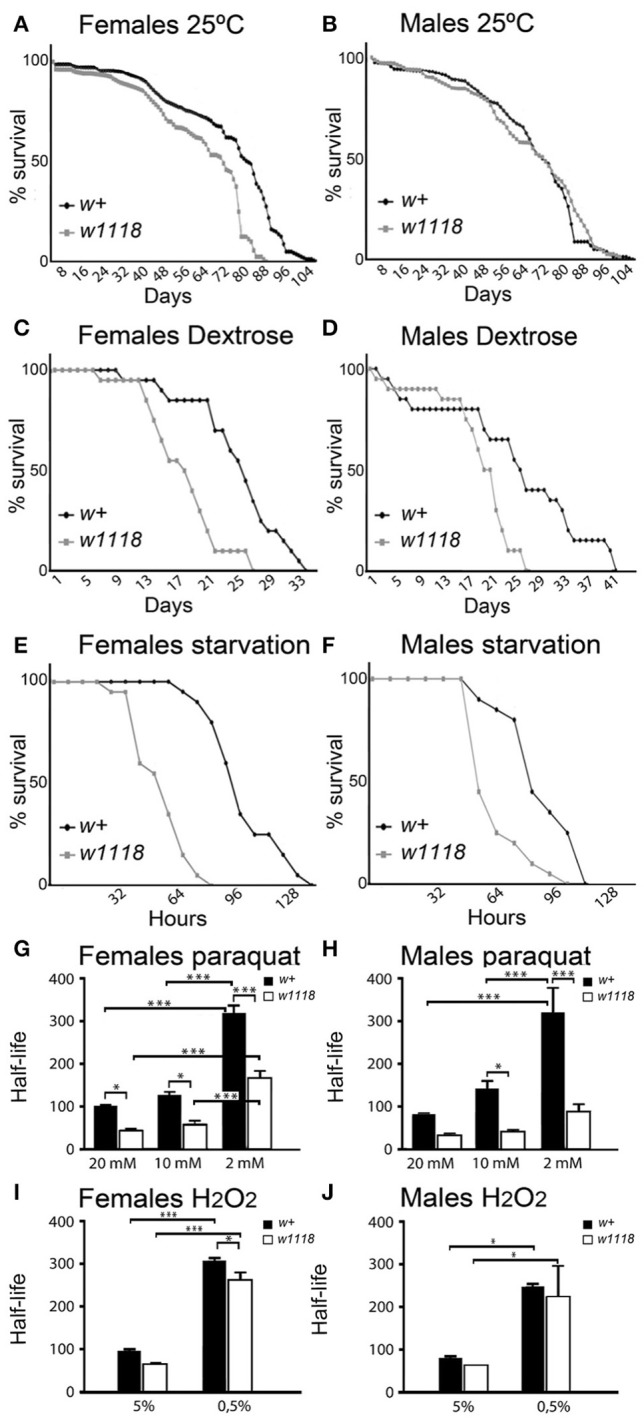
*w*^*1118*^ mutants have reduced life span and are more sensitive to stress treatments. Graphical representation of total life span **(A–F)** or half-life, i. e., the time corresponding to 50% of survival **(G–J)** of *w*^+^ (Oregon strain) vs. *w*^*1118*^ flies, kept either under standard conditions at 25°C **(A,B)** or under stress conditions: dextrose treatment **(C,D)**, starvation **(E,F)**, or oxidative stress caused by dietary administration of paraquat **(G,H)** or H_2_O_2_ **(I,J)**. In **A–F**, the total number of survivor flies per day was plotted per experimental condition. **(G–J)** show half-life plots discriminated per genotype, sex and oxidative agent concentration (20, 10, and 2 for paraquat treatment in **G,H**; 5% and 0.5% for H_2_O_2_ treatment in **I,J**). Significance was calculated using Two-Way ANOVA, Bonferroni *post-hoc* test: ^*^p < 0.05; ^***^p < 0.001. The bars indicate s.e.m.

Shorter life span, relative to wild-type strains, is another key feature of all neurodegenerative phenotypes in *Drosophila* (Lessing and Bonini, 2009; Hirth, 2010; Jaiswal et al., 2012). We performed life span experiments on female and male flies of *w*^*1118*^ and *w*^+^ strains at standard temperature (25°C), under normal raising conditions -ie. without stressors- (Figures 4A,B) or under different forms of stress (Figures 4C–J).

We found that *w*^−^ mutants had shorter life span relative to *w*^+^ at most conditions tested. Under normal raising conditions at 25°C, we observed a significant life shortening in *w*^*1118*^ females compare to *w*^+^ females (*w*^*1118*^ vs. *w*^+^ half-life was 47 vs. 65 days, *p* < 0.01; Figure 4A). In males, *w*^*1118*^ vs. *w*^+^ half-life was 43 vs. 49 days (*p* > 0.05; Figure 4B).

We then tested the resistance of *Drosophila w*^−^ mutants to two different challenges, starvation and sugar-enriched diet (reviewed in Ristow and Schmeisser, 2011). Female or male flies from *w*^+^ and *w*^*1118*^ strains were kept under standard laboratory conditions except that their food was either absent (starvation) or enriched with dextrose (Figures 4C–F). Our results confirmed previous reports in which, irrespective of their genotypes and sexes, flies kept under a high-sugar diet have shorter life span than flies raised on standard diet (compare Figure 4C to Figure 4A and Figure 4D to Figure 4B). Most importantly, we observed that *w*^−^ mutants were significantly less resistant to a sugar-enriched diet than *w*^+^ flies. In females *w*^*1118*^ vs. *w*^+^ half-life was 18 vs. 26 days (*p* < 0.0001; Figure 4C) while in males was 20 vs. 26 days (*p* < 0.0001; Figure 4D). *w*^*1118*^ flies were also significantly less resistant to starvation than *w*^+^ flies. In females, *w*^*1118*^ vs. *w*^+^ half-life was 64 vs. 104 h (*p* < 0.0001; Figure 4E) while in males was 56 vs. 80 h (*p* < 0.0001; Figure 4F).

A strong association between neurodegenerative pathologies and oxidative stress is well-documented in humans and other mammals (Yan et al., 2013; Cobb and Cole, 2015; Kim et al., 2015) as well as in flies (Gruenewald et al., 2009). We compared the resistance of *Drosophila w*^1118^ mutants to oxidative stress relative to *w*^+^ flies, with two widely used oxidative agents at various concentrations (Figures 4G–J). Flies of each genotype were kept under standard laboratory conditions except that their diet contained either paraquat (Figures 4G,H) or hydrogen peroxide (Figures 4I,J). Both treatments are reported to induce oxidative stress and to have consequences at the transcriptional level (Zou et al., 2000; Landis et al., 2004, 2012; Brown et al., 2009).

We found that *w*^*1118*^ mutants of both sexes were significantly less resistant to paraquat than *w*^+^ flies. After paraquat treatment, the half-life of females was 56 h in *w*^*1118*^ relative to 112 h in *w*^+^ (*p* < 0.05) at 20 mM; 72 vs. 128 h (*p* < 0.05) at 10 mM and 192 vs. 320 h (*p* < 0.001) at 2 mM (Figure 4G). In males, half-life was 40 vs. 88 h (*p* > 0.05) at 20 mM; 56 vs. 168 h (*p* < 0.05) at 10 mM and 80 vs. 296 h (*p* < 0.001) at 2 mM (Figure 4H). *w*^*1118*^ females were also significantly less resistant to hydrogen peroxide than *w*^+^ at the 0,5% concentration (*w*^*1118*^ vs. *w*^+^: 264 vs. 320 h (*p* < 0.05; Figure 4I).

### Expression of the *mini-white^+^* transgene in *white* mutants rescues the retinal-degeneration phenotype

Most of the transgenic stocks generated in a *w*^*1118*^ mutant background carry a form of the gene *w* (*mini-w*^+^) that recuperates the eye color to a certain extent.

**Figure 5:**
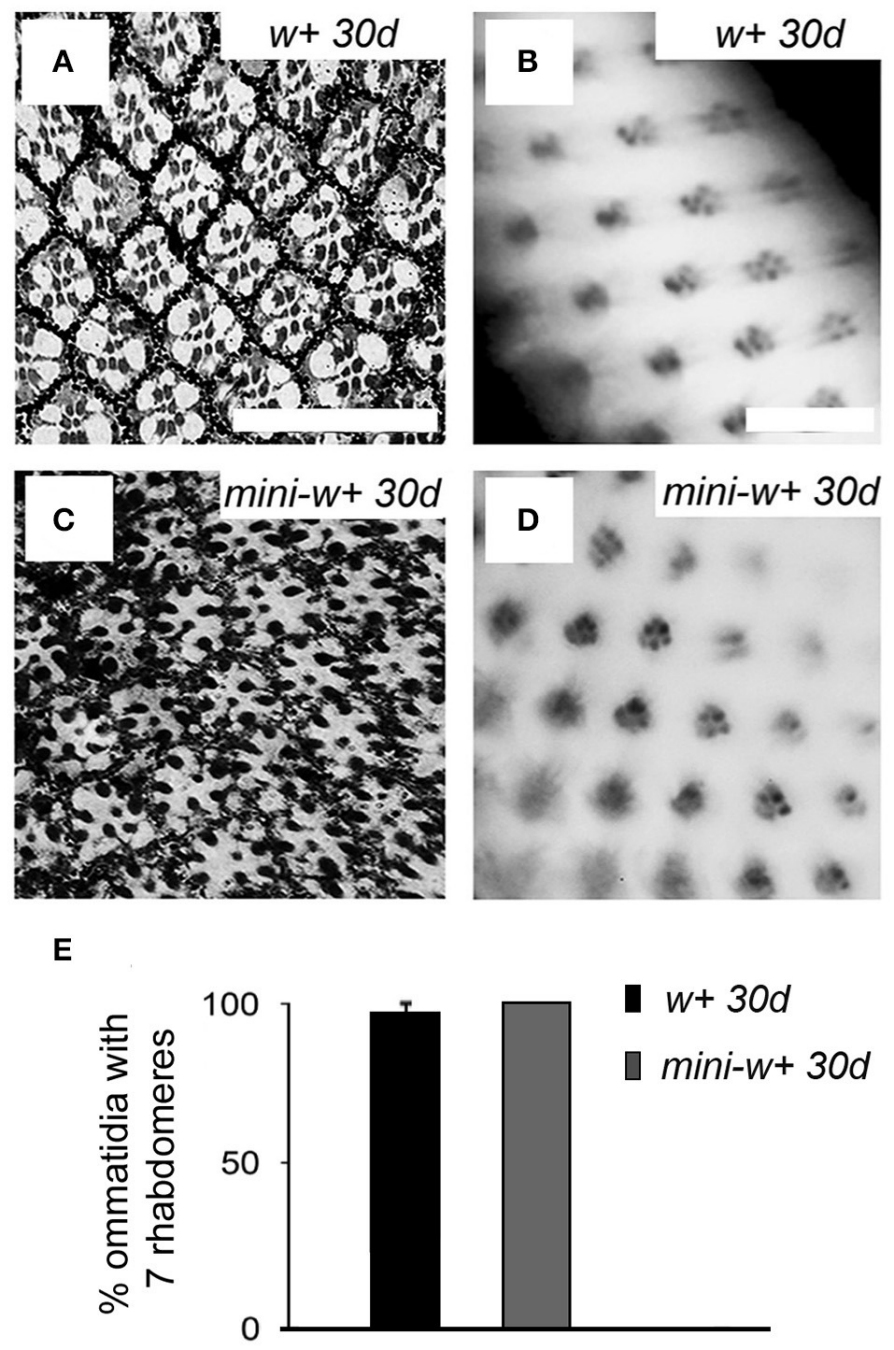
Expression of the *mini-white*^+^ transgene in *w*^*1118*^ mutants rescues the retinal degeneration phenotype. Histological microscopy sections of the retina from 30 days-old *w*^+^ flies from the Vallecas strain **(A)** and transgenic flies expressing *mini-w*^+^ in a *w*^*1118*^ null genetic background **(C)** showed a *w*^+^-like organization as neither missing ommatidia nor rhabdomeres was observed (compare with *w*^−^ mutants, Figures 1F,G). **(B,D)** Images of the retina obtained by the method of optical neutralization of the cornea in *w*^+^ **(B)** and transgenic flies expressing *mini-w*^+^ **(D)**, showing that the expression of *mini-w*^+^ in a *w*^−^ background rescues the phenotype. Scale bars represent 20 μm. **(E)** Graphical representation of the percentage of ommatidia with seven rhabdomeres in 30 days-old flies from *w*^+^ and *mini-w*^+^ strains showing that there are no significant differences in this parameter between both genotypes (*w*^+^ 30 d vs. *mini-w*^+^ 30 d, Mann–Whitney *U*-test, *p* >0.05).

Here we found that expression of a *mini-w*^+^ *Drosophila* transgene in *w*^*1118*^ flies rescued not only the eye pigmentation phenotype, but also the retinal-degeneration phenotype that we had detected in *w*^−^ mutant flies (Figure 5). Neither missing rhabdomeres nor lacunae (missing ommatidia) were observed in 30 days-old flies expressing *mini-w*^+^ across the eye in a *w*^*1118*^ null genetic background (compare Figures 5A,B with Figures 5C,D; see Figure 5E, percentage of ommatidia with seven rhabdomeres).

## Discussion

The most important contribution of this study is the demonstration that the *w*^−^ mutant strain most widely used in *D. melanogaster* research (*w*^*1118*^) suffers from a degenerative pathology that worsens with age. This expands on the concerns raised by a previous report that this same mutation exacerbates the neurodegenerative phenotype induced by *tau* expression (Ambegaokar and Jackson, 2010). Taken together, these concerns underline that caution is needed when interpreting and drawing conclusions from hundreds of previous experiments in which *w*^*1118*^ flies were used as transgenic tools or as substitute for *w*^+^ controls.

Our results indicate that the defects caused by the mutations in *w*^*1118*^ are very mild at the beginning of adult life, i.e., the age when these mutant flies are most frequently used as controls. Thus, the potentially negative consequences of using *w*^*1118*^ as the only experimental controls could be perhaps reduced or abolished if researchers refrain from using mutant flies older than 4–5 days and include a *w*^+^ stock as wild-type control if necessary. Nevertheless, even *w*^−^ mutant flies of this young age showed symptoms (to some degree) in several of the assays presented here, as well as in other assays reviewed in the section Introduction.

It appears clear that expression of the *mini-w*^+^ gene in a *w*^*1118*^ genetic background provides almost complete rescue of the *w* mutant phenotype of retinal histology. This, combined with the observation of the same phenotype -retinal degeneration- in a second allele (*w*^*1*^) demonstrate that is the lack of *w* expression in the fly eye which is responsible for this pathology. This phenotype is a stronger in *w*^*1118*^ than in *w*^*1*^, which is consistent with the nature of both mutations. The allele *w*^*1118*^ is defined as a spontaneous null allele caused by the deletion of the 5′ half of *w* gene (http://flybase.org/reports/FBal0018186.html) while *w*^*1*^ allele is a spontaneous insertion of a Doc transposable element close to the site of transcription initiation of the *w* gene, while the coding region remains unaffected (http://flybase.org/reports/FBal0018074.html). Therefore, *w*^*1*^ mutation has been considered as hypomorphic (Driver et al., 1989; Lloyd et al., 2002). This could be the reason why the phenotype is milder in *w*^*1*^ with respect to *w*^*1118*^. The expression of *mini-w*^+^ in a *w*^*1118*^ background would not necessarily rescue all other aspects of the mutant phenotype (i.e., behavioral deficiencies as the one demonstrated here in the climbing ability). The mechanism by which the absence of White affects behavioral phenotypes could be rather complex as *w* is expressed in tissues other than eyes. *mini-w*^+^ stocks have a range of eye coloration depending on the position of the *mini-w*^+^ insertion into the genome (reviewed by Silicheva et al., 2010). It has been suggested that the *w* promoter might function as an “enhancer trap”, 5′ and 3′ enhancers stimulating *mini-w*^+^ transcription. The sensitivity of *mini-w*^+^ to chromosomal position effects could perhaps also explain the failure to recover all behavioral phenotypes, through the positive or negative effect of external enhancers. Krstic and co-workers (Krstic et al., 2013) found that the *w* mutation affects courtship behavior. The authors showed that *w*^*1118*^ males kept in darkness lose their preference for females. Interestingly, this behavioral phenotype was not rescued by the expression of a *mini-w*^+^. The authors proposed that, although *mini-w*^+^ is fully expressed in the eye of transgenic flies, it lacks the enhancers required for its expression in the central and peripheral nervous systems. Accordingly, they suggested caution when drawing conclusions on behavioral experiments based on *w*^−^ mutants.

It was already reported that constant illumination causes retinal degeneration and malfunction in *Drosophila w*^−^ mutants (Shoup, 1966; Wu and Wong, 1977; Schraermeyer and Dohms, 1993; Lee and Montell, 2004; Bulgakova et al., 2010; Belušič, 2011). Here we demonstrate for the first time that retinal degeneration develops in *w*^−^ mutants even when the flies are maintained under standard light:dark cycles and, perhaps more importantly, we reveal the progressive nature of this pathology. Using electron microscopy and histochemistry, Shoup (Shoup, 1966) had shown that the retina of *w*^−^ mutant flies exposed to constant illumination produced atypical “lysosome-like” organelles. Extending this observation, it was later on demonstrated that these organelles were of lysosomal origin and the possibility that these abnormal lysosomes will result from “abnormal degradation of the photosensory membrane” was suggested (Schraermeyer and Dohms, 1993). Subsequently, it was demonstrated that autophagy of activated rhodopsin has a neuroprotective function against the light-induced degeneration of the retina in *Drosophila* (Midorikawa et al., 2010). Today it is well established that abnormalities in the lysosomal/autophagy pathway are functionally related to several neurodegenerative pathologies (Nixon, 2013; Ingemann and Kirkegaard, 2014; Fraldi et al., 2016). Moreover, the*w*^*1118*^ mutation aggravates the retinal degeneration caused by transgenic expression of human Tau, which is also possibly explained by malfunction of the lysosomal/autophagy pathway (Ambegaokar and Jackson, 2010). We believe that if the White protein is required for the normal function of lysosomes, the *w*^*1118*^ mutation could have negative consequences for several biological functions in a variety of tissues, including the maintenance of the retina, the control of protein turnover and more in general, the fly's resistance to stress challenges. Moreover, we believe that beyond being used as controls for other genotypes in neurodegeneration experiments, *w*^*1118*^ flies themselves could become an important tool for the study of the functional relationship between the lysosomal pathway, autophagy, and the formation of nanofilaments during neurodegeneration.

The enlarged receptor potential seen here in mutant retinas is consistent with previous studies (Belušič, 2011) and its subsequent reduction in mutants of older age is consistent with the progressive character of the retinal degeneration documented here. A likely explanation is that in the young *w*^−^ mutant flies, the absence of eye pigment causes a massive response because many more rhabdomeres are exposed to light than in normal flies, because of the lack of pigment around them. Twenty-five days later, that initial response is much reduced because of the progressive loss of photoreceptors and the smaller size of the rhabdomeres (the light-sensitive components) of the remaining photoreceptors.

As mentioned in the Introduction, a variety of behavioral assays conducted by others have revealed other neurological defects in *w*^*1118*^ flies, indicating that the protein White probably has other important functions in the brain of *Drosophila* flies. Part of those problems can relate to the abnormally low levels of serotonin and dopamine (Borycz et al., 2008; Sitaraman et al., 2008). Our observation that *Drosophila w*^−^ mutants have low resistance to stress induced by paraquat, hydrogen peroxide, high dextrose diet, or starvation, indicates a wider problem, which reflects perhaps one or more functions of White in tissues other than the eye and brain.

Besides the eye, where *Drosophila w* gene is expressed at relatively high levels, and the brain where it is expressed at low levels, *w* is expressed at very high levels in the excretory system and at relatively low levels in several other tissues of the fly (Chintapalli et al., 2007). Experimental evidence indicates that at least in the excretory organs White acts as a transporter of cyclic GMP and, therefore, might be important in this and other tissues for the regulation of several biological functions through cyclic GMP signaling (Evans et al., 2008). This suggests that a loss of function in the *w* gene would result in a compound phenotype with deeper and more widespread physiological consequences than assumed so far and is in agreement with abundant experimental evidence indicating that the excretory tubules are important for the organism's response to stress (Davies et al., 2014).

As a concluding remark, we encourage researchers to always include *w*^+^ controls and to discriminate sex and age of individuals in *Drosophila* experiments where *w*^−^ mutants are used alone or as a control for transgenic strains with a *w*^*1118*^ genetic background.

## Ethics statements

We use *D. melanogaster* as a model animal for this study, in accordance, the study was exempt from ethical approval procedures.

## Author contributions

RC: conceptualization; MF, RB, RC: designed the methodology; MF, RB, RC: performed the formal analysis; MF, CP, MM, SR, PA, AC, RB, RC: performed experiments; RC: wrote the original draft; MF, RB, RC: wrote, reviewed and edited the published version; MF, PA, AC, RB, RC: designed figures; RB, RC are responsible for funding acquisition, supervision, and administration of the project.

### Conflict of interest statement

The authors declare that the research was conducted in the absence of any commercial or financial relationships that could be construed as a potential conflict of interest.
